# Nurses’ perspectives on the use of telemonitoring in the management of people with diabetes and hypertension

**DOI:** 10.1590/0034-7167-2023-0481

**Published:** 2025-01-13

**Authors:** Camila Wohlenberg Camparoto, Maria do Carmo Fernandez Lourenço Haddad, Elen Ferraz Teston, Pamela dos Reis, Sonia Silva Marcon

**Affiliations:** I Universidade Estadual de Maringá. Maringá, Paraná, Brazil; II Universidade Estadual de Londrina. Londrina, Paraná, Brazil; III Universidade Federal do Mato Grosso do Sul. Campo Grande, Mato Grosso do Sul, Brazil; IV Universidade do Vale do Itajaí. Itajaí, Santa Catarina, Brazil

**Keywords:** Patient Monitoring, Health Education, Public Health, Nurse, Delivery of Health Care, Telemonitoreo, Hipertensión Atención Primaria de Salud, Enfermeros, Enfermedad Crónica

## Abstract

**Objectives:**

to understand the perspective of nurses on the use of telemonitoring in the management of people with type 2 diabetes *mellitus* and arterial hypertension in primary care.

**Methods:**

this qualitative research involved sixteen nurses from eight municipalities in Paraná. Data were collected between November 2022 and January 2023 through inperson or remote interviews, which were audio-recorded and subjected to content analysis.

**Results:**

according to the nurses, telemonitoring enhances users’ knowledge about these conditions, communication and connection with the team, and productivity. However, the lack of electronic resources and equipment, high staff turnover, low user adherence, and the limited availability of professional time present significant challenges.

**Final Considerations:**

the effective implementation and operation of telemonitoring in the management of people with diabetes and hypertension involve both potential benefits and barriers. It is essential to have the availability of human and technological resources, managerial support, and the commitment of professionals and users.

## RESUMEN

**Objetivos:**

comprender la perspectiva de los enfermeros sobre el uso del telemonitoreo en el acompañamiento de personas con diabetes *mellitus* tipo 2 e hipertensión arterial en la atención primaria.

**Métodos:**

investigación cualitativa con dieciséis enfermeros de ocho municipios de Paraná. Los datos fueron recolectados entre noviembre de 2022 y enero de 2023, mediante entrevistas presenciales o remotas, grabadas en audio y sometidas a análisis de contenido.

**Resultados:**

para los enfermeros, el telemonitoreo favorece el conocimiento del usuario, comunicación y el vínculo con el equipo, y su productividad; sin embargo, falta de recursos electrónicos y equipos, rotación del personal, baja adhesión de los usuarios y falta de tiempo de los profesionales constituyen desafíos.

**Consideraciones Finales:**

implementación y operacionalización efectiva del telemonitoreo en el seguimiento de personas con diabetes e hipertensión implican potencialidades y barreras, siendo fundamental la disponibilidad de recursos humanos y tecnológicos, apoyo de Gestión, disposición de profesionales y usuarios.

## INTRODUCTION

The epidemiological, nutritional, demographic, and technological transitions have triggered an increase in Non-Communicable Diseases (NCDs), resulting in high costs for healthcare systems and negative impacts on quality of life. In light of this, there is an urgent need to find and prioritize cost-effective strategies applicable to the context of primary care, focusing on the prevention and management of these diseases^(1)^.

According to the World Health Organization (WHO), Chronic Diseases (CDs) are responsible for 41 million deaths each year. They constitute a global problem, as only 25% of people with CDs receive healthcare services, and only half of these individuals achieve the intended clinical care goals. This outcome is largely due to inadequate access to healthcare and difficulties in self-managing one’s health condition^(2)^.

Although the purpose of the Unified Health System (in Portuguese SUS) since its creation has been the reorganization of the country’s healthcare model, significant challenges persist, especially regarding the proper management of chronic conditions and the engagement of individuals in self-care activities. This situation challenges researchers, healthcare services, and health professionals to constantly seek care models that can provide better outcomes^(3)^.

Considering this perspective, the Chronic Conditions Care Model (CCM) was proposed in 2011, aiming for continuous and proactive care within the SUS^(4)^. Its purpose is to help reduce the worsening and mortality of conditions that are sensitive to Primary Health Care (PHC), such as chronic diseases^(5)^. PHC serves as the main gateway to the healthcare system and prioritizes disease prevention and health promotion without neglecting care services^(6)^.

The CCM, in turn, is a care model that, to be effectively incorporated, requires professional training, as it involves changes in the care process for people with chronic conditions^(7)^. Investing in on-the-job training enables the appropriate performance of health professionals and managers working in PHC, particularly in the care provided by Family Health Strategy (FHS) teams. Adequate care can help reduce hospitalizations and deaths resulting from the worsening of chronic conditions^(8)^.

Some strategies have proven successful in the prevention and management of Hypertension (HTN) and Diabetes Mellitus (DM), especially type 2 (DM2), whose control is closely related to behavior and lifestyle changes. These include care protocols, teamwork—with adequate communication among team members—follow-up and monitoring of chronic conditions, health promotion activities, the identification of new cases, and people at higher risk, among others^(8)^. However, the challenge lies in the management and maintenance of these actions^(9)^.

Monitoring in the healthcare sector gained prominence during the COVID-19 pandemic, as the health crisis, marked by a lack of hospital beds and social isolation, required the monitoring of reported cases^(10)^. In this scenario, people with chronic conditions were considered highly vulnerable and were therefore advised to avoid healthcare services, while still needing to have their clinical conditions minimally monitored. Thus, it was realized that a significant portion of these individuals, provided it was done in a planned manner, could be monitored remotely through telemonitoring (TM) without harm to their health.

It is important to highlight that, in PHC, TM involves using methods that allow healthcare professionals to monitor clinical parameters and obtain relevant information about users’ health conditions, whether through smartphone apps or other platforms, emails and SMS, or phone calls^(11)^. Access to these technologies and resources facilitates the remote monitoring of the progress and effectiveness of treatment by both the healthcare team and the user and/or their families^(12)^.

Even before the pandemic, based on studies conducted in other countries, TM for chronic conditions was already being discussed in Brazil, emphasizing the possibility of cost reduction, in addition to other benefits such as reduced emergency room visits, acute hospitalizations, rehospitalizations, and time spent on home care, among others^(13)^. Indeed, some private health insurance companies were already implementing these actions, considering the positive results for the user^(14,15)^ and the institution^(16)^ reported in the literature.

However, in Brazil, studies on the use of this assistive technology are still incipient and isolated. TM has been used to assess effects on obesity control, with one study highlighting the ease of user access to healthcare professionals^(17)^, and another finding a reduction in the risk of metabolic syndrome^(18)^. Internationally, studies confirm the benefits for people with various chronic conditions^(11,19)^. Research conducted in Denmark, for example, found that, from the perspective of nurses, doctors, and patients with chronic diseases, although it was challenging to introduce a technology like TM into everyday care, it was able to improve patients’ health conditions^(20)^.

A review study of randomized clinical trials addressing interventions focused on chronic disease self-management indicated that TM, among other strategies, can support change and sustain long-term efforts to halt disease progression and encourage health behaviors, improving users’ quality of life and reducing healthcare service use^(21)^. Remote educational interventions have also shown a positive influence on health literacy and knowledge about hypertension, even impacting lifestyle choices. These aspects highlight TM’s potential as a form of health monitoring and promotion, supporting disease management^(22)^. However, a study investigating PHC nurses’willingness to use TM as an auxiliary tool in monitoring people with DM and/or HTN found that willingness was lower among older nurses, those with longer professional training and experience in PHC, and those who did not believe that this care strategy facilitates communication with patients^(23)^.

In this context, the question arises: Is the use of TM as an auxiliary strategy viable for monitoring people with chronic conditions in PHC?

## OBJECTIVES

To understand the perspective of nurses on the use of telemonitoring (TM) in the management of people with type 2 diabetes mellitus and arterial hypertension in PHC.

## METHODS

### Ethical Aspects

In the development of the study, all ethical guidelines established by Resolutions 466/2012 and 510/2016 of the National Health Council and the Guidelines for Research Procedures at Any Stage in Virtual Environments – CONEP/2021 were followed. The project was approved by the Research Ethics Committee with Human Subjects of the signing institution. Participants agreed to participate in the study by signing the Informed Consent Form (ICF). In the case of remote interviews, the ICF was sent via an online form. To ensure anonymity in the presentation of results, excerpts from the interviews are identified with the letter E, indicating the interviewee, followed by a number indicating the order of the interview, e.g., E1.

### Theoretical-Methodological Framework

The conceptual basis was the CCM, structured on the principles of the SUS. It proposes the organization of healthcare activities for the population based on five levels of intervention, according to the determinants and needs of the population^(4)^.

### Type of Study

This is a descriptive, exploratory study of a qualitative nature, not intended to produce generalizable results. The Consolidated Criteria for Reporting Qualitative Research (COREQ) were used to guide the research report^(24)^.

### Methodological Procedures

The informants of the study were nurses working in the PHC of municipalities that are part of the 15th Health Region of Paraná, who participated in workshops to raise awareness about the use of TM in the management of people with chronic conditions. At least three attempts to contact the workshop participants were made to invite them to participate in the study and to schedule interviews if they agreed. All those who accepted were included.

### Study Setting

The study setting included the municipalities that are part of the 15th Health Region (HR) of Paraná. All nurses working in the PHC of the 30 municipalities in this HR were invited to participate in an intervention, but only 16, from eight municipalities, actually participated. The proposed intervention involved preliminary readings, practical activities—such as testing the developed instruments—and five awareness-raising workshops on the use of TM in the management of people with chronic conditions. The workshops lasted 60 to 90 minutes and were held weekly, from May to June 2020, remotely, on days and times that the participants found most convenient.

In the meeting prior to the start of the intervention, the nurses were invited to report and reflect on the characteristics and outcomes of the care provided to people with HTN and/or DM in their municipalities. The group concluded that the care was fragmented and focused on the complaints/conditions presented by the users, based on the recommendations contained in the *Linha Guia of the State of Paraná* ^(25)^.

After sharing experiences and discussing possible care approaches guided by various readings, the group collaboratively developed a TM Protocol (telephone calls and WhatsApp messages) for monitoring people with HTN and/or DM in PHC, as well as some tools to guide care and its documentation. The developed protocol includes the following steps: 1) Identify users eligible to participate; 2) Determine the user’s risk level; 3) Conduct an in-person nursing consultation to identify problems, set goals to be achieved, and establish the frequency of phone calls; 4) Develop a care plan; 5) Make telecontacts to follow up on the care plan; and 6) Evaluate the achievement of goals.

Finally, the group decided that the protocol should be tested, and for this purpose, each nurse was instructed to implement TM for a period of 12 months with 30 users registered in their Basic Health Unit (UBS in Portuguese).

### Data Collection

Data were collected from November 2022 to January 2023, 17 months after the workshops, through semi-structured interviews that were audio-recorded with consent and conducted remotely or in person, according to the participant’s choice, on the day, time, and place of their preference.

The interviews lasted an average of 18 minutes and were guided by a semi-structured script, consisting of two parts: the first with questions aimed at the sociodemographic characterization of the participants, and the second composed of the main question: “What is your opinion about TM today?”. Some supporting questions were used, such as: “Were you able to implement it? Tell me more about that”; “How was your experience with TM?”; and “What were your difficulties?”.

All interviews were conducted by the same researcher (a nurse and master’s student in nursing, with some experience in the collection and analysis of qualitative data from participating in other research projects), who had no relationship with the study participants.

### Data Analysis

For data processing, the interviews were fully transcribed and subjected to content analysis using a thematic approach, which follows three stages^(26)^. In the pre-analysis stage, the transcribed material was organized and read repeatedly, with the identification of recording units. In the material exploration stage, coding was conducted, and provisional categories were identified, with terms derived from the data readings. Finally, in the data treatment and interpretation stage, codes were regrouped based on similarities, confirming the two provisional categories, followed by inferences about their relationships and interpretation in light of the CCM.

## RESULTS

Of the 16 nurses participating in the study, only one was male. All had been working in PHC for more than five years (with an average of 15 years), ten had a specialization, and two had a master’s degree. From the data analysis, two categories emerged, which will be presented below.

### Potential of telemonitoring in Monitoring Patients with Hypertension and Diabetes

Among the benefits of using TM, nurses highlighted the possibility of providing users with more information about their health condition, which they believe enhances the quality of care.

*They clear up a lot of doubts, they end up interacting more [...] and we already provide the guidance, then they give feedback and follow the instructions. (E5)*

*Most users with chronic diseases have a low level of knowledge [...] sometimes [...] they don’t know what they can eat, the risks of a [...] small injury, that the injury may take a long time to heal [...] and they end up developing more serious problems, which require more resources [...] a higher level of care [...] with more costs* [...]*. It facilitates contact [...] with that, I think their quality of life improves a lot, really a lot. (E10)*

Remote contact facilitates communication and access to information.

*Those younger people who work and have WhatsApp, it makes [contact] much easier. (E2)*

*Not having to go to the unit and at the same time giving a reminder, to keep them remembering what they need to do* [...]*. (E11)*

Furthermore, they recognize the importance of this resource as a strategy to establish, and even strengthen, the bond with users, especially those who see telecontacts as a more individualized form of care that makes them feel more valued.

*It’s a way to have quicker and more frequent, continuous contact with the patient [...] with the community [...] patients receive it very well, they even like this more individualized attention... as if they felt more valued. (E5)*

*It’s the possibility of a bond, you start forming it in the first consultation. And then, you maintain this bond with the patients through monitoring, some feel valued by having a healthcare professional calling them* [...]*, giving guidance and asking how they are doing* [...]*. (E9)*

They highlighted that TM also brings benefits to the service, making it more productive by enabling care for a larger number of users in the same amount of time.

*In the time it would take to visit five users, you can, with TM, care for ten users. (E10)*

They also emphasized the important role of TM during the pandemic, due to the difficulty patients had in traveling to the health service.

*I think it was very useful during the pandemic period [...] it was possible to monitor patients who stayed away from the units* [...]*. So [...] TM [...] allowed us to stay a little closer to these users* [...]*. (E10)*

*During the pandemic [...] patients weren’t coming to the unit and it was a way for us to communicate, to know if they were okay, if they were taking their medication, to know how their blood pressure or blood glucose, for those who had devices at home, was going, if they were controlling it* [...]*. (E13)*

*Talking to the patient and knowing a little more about their daily life [...] regarding eating habits, you could notice a lot during those consultations. I got to know my patients better, even those I had daily contact with at the UBS* [...]*. (E11)*

The reports included in this category highlight the potential of TM from the nurses’ perspective, emphasizing the transmission of knowledge about the disease and its treatment, ease of communication, strengthening of bonds, enhancement of care quality, and team productivity.

### Challenges encountered in implementing telemonitoring

Nurses reported that one of the barriers they faced in implementing TM was the lack of specific equipment, such as telephone lines or mobile devices.

*I had to use my personal cell phone [...] and that ends up being a problem* [...]*. To use the landline, we had to use one line, and there are only a few lines. (E10)*

*We had difficulty with calls because there’s only one phone line, and here we have two teams, so that made it difficult. (E12)*

*In terms of phones [...] if we use our own, we can speed things up. Relying on the UBS phone, I find it a bit more complicated,* [...]*. It’s a barrier. (E4)*

*It’s only been a month and a half since they installed WhatsApp, because before that, we didn’t have it [...] I think this technological aspect helps. (E2)*

*One issue we couldn’t resolve was getting cell phones [...] because the municipality didn’t commit to purchasing them. (E10)*

This is because messaging apps (like WhatsApp) were identified as the first option for establishing contact with users.

*We don’t have WhatsApp, which could have been a way to communicate with patients* [...]*. So it’s only the landline. (E6)*

*We didn’t have WhatsApp, so the little we managed to communicate was using the cell phones of the CHWs [Community Health Workers], and that was very difficult because it wasn’t specific to TM; it was general WhatsApp* [...]*. (E3)*

However, according to the nurses, some users expressed dissatisfaction with the service when contact was made through apps, or they did not respond to the messages.

*They weren’t satisfied with guidance only through WhatsApp [...] they wanted to come in, even though we gave them the same reminders* [...]*. (E8)*

They also discussed barriers related to the physical space for conducting the in-person consultation, which precedes TM.

*The difficulty is that the physical space was limited [...] we don’t have the infrastructure, we started having [...] certain conflicts. (E9)*

*I don’t have a specific nursing office [...] where I could comfortably do this. So usually, it’s the assistant and the nurse together [...] for me, it’s quite [...] complicated. Our unit’s structure can’t handle*

[...] *so many people, so many appointments that we have. (E16)*

The fact that the team was incomplete was also pointed out as a factor leading to overloads, which compromised the implementation of TM.

*We are without a nurse [...] I think the main problem is the lack of professionals [...] it’s difficult because we know it’s humanly impossible* [...]*. (E12)*

*We were without a doctor in the unit, we had many complications*

[...] *and we couldn’t carry out the program as we wanted* [...]*. (E14)*

Even the UBS that had the necessary technological and structural resources faced difficulties in implementing TM. One of the challenges mentioned was the need for users to travel to the UBS without a specific complaint and the time spent on the initial nursing consultation.

*I think the biggest difficulty was getting them to come to the UBS for the nursing consultation.* (E11)

*Sometimes the difficulty is in getting the patient to come [...] because they live a little far away, or because they have trouble walking and don’t have, a [...] a means of transportation, a car, or someone to bring them* [...]. (E1)

*It was complicated to register them, because it’s a longer consultation, where we have to assess the patient, and sometimes they want to leave or avoid the consultation.* (E2)

Another aspect mentioned was the fact that some users did not adhere to the purposes of this care model, which focuses on self-care and continuous monitoring by the professional.

*Those patients who really needed it, they adhered, but those who were more controlled, they didn’t seem to care as much.* (E2)

Some comments indicated that part of the users showed resistance to changes in the care model.

*The biggest resistance is from the patient in changing the care model, because while everything was stopped due to the pandemic, they accepted it. Now that vaccination has started to balance things out, they’ve lost the fear of coming to the UBS, so they prefer to come to the office [...] rather than by phone.* (E2)

*People still have a lot of difficulty, difficulty expressing themselves, which can’t be replaced by a face-to-face conversation. It’s the patients themselves [...] the difficulty in expressing what they feel [...] what’s going on, it’s the difficulty with the means of communication.* (E3)

The limitation that some users had in using technology was another barrier mentioned.

*What didn’t help is [...] they didn’t have much command of [...] the technology.* (E13)

*The issue of guidance [...] I think sometimes you’re on the phone giving guidance [...] especially with the elderly, they get very confused over the phone.* (E4)

*Some have difficulty even understanding, we say one thing, and they understand another* [...]. (E12)

As well as the fact that users do not answer phone calls or even respond to written messages on WhatsApp.

*Most didn’t answer, I even formulated and sent it on WhatsApp [...] I identified myself [...] but many didn’t respond [...] and most would reply kind of [...] evasively.* (E2)

*They don’t answer the call, they don’t respond, they see the message, read it, but don’t want to reply* [...]. (E9)

*Sometimes patients don’t answer the phone, you try once, try twice, try three times... and they don’t answer* [...]. (E14)

They also reported the importance of the bond between professionals and users for adherence, but also the difficulties encountered in creating and maintaining this bond in the face of high staff turnover in the services.

*Patients who had a stronger bond were more willing to embrace the idea. If they have a stronger bond with the professional, adherence is easier* [...]*. (E2)*

*The turnover of professionals ended up breaking all the bonds that had been established at the beginning, and, like it or not, the user ends up distancing themselves, not wanting to participate because they don’t have a bond. (E12)*

Finally, they highlighted the overload of activities, mainly due to conducting the Nursing Consultation, which, although it is a nurse’s responsibility and one of the priority actions in the context of PHC, was not routinely performed in the services studied.

*The consultation itself for the nurse to conduct, I think it’s quite complicated* [...]*. We already have a lot going on day to day. (E4)*

*Regarding the nursing consultation, the physical exam itself, we are required to do it, but at the moment it’s not being done, there’s no way* [...]*. (E6)*

*If I say I had enough time, I didn’t* [...]*. At the beginning, it was more difficult [...] it took a lot of time [...] to fill out that form. We weren’t completing it fully. I think the biggest problem was the lack of time* [...]*. (E8)*

*It’s hard to do because the teams are very overloaded, with a lot to do, many demands, post-pandemic, a lot to organize, reorganize* [...]*. (E9)*

The reports in this category show that the professionals are interested in conducting TM with the users registered in their team, but they face various difficulties related to physical structure, limited electronic resources, staff turnover, user adherence, and lack of time to carry out related activities.

## DISCUSSION

This study allowed us to understand that nurses see potential in using TM as an auxiliary strategy to monitor people with chronic conditions (CC) in PHC, and the participants expressed interest in using it. However, they emphasized that the ability to implement changes in care through new technologies is related to basic aspects, such as the availability of electronic communication equipment, physical infrastructure, and human resources.

Chronic conditions such as HTN and DM are managed by teams working in PHC, responsible for providing care aimed at health promotion and disease prevention in a continuous, comprehensive, and coordinated manner. However, the nurses in this study pointed out that management is based on a biomedical and curative care model, which has been highlighted as a barrier to offering care that involves the user in decision-making about their health condition to reduce complications^(3)^, as proposed by the CCM.

In this regard, they considered the possibility of using a strategy to monitor PHC users in an individualized manner as positive. This finding corroborates the results of a study conducted in Santa Catarina^(12)^, which, although it identified the need for better planning with more regular monitoring and the establishment of specific goals in the care plan, showed that monitoring users with HTN and DM, especially those who are insulin-dependent, had a positive impact on reducing complications, avoiding hospitalizations and unnecessary consultations. Therefore, the nurses identified that an individualized approach to users was beneficial^(12)^.

In healthcare, Information and Communication Technologies (ICTs) are used to help people self-manage chronic diseases and have shown positive effects^(27)^. The advantages of using interventions with ICTs include their non-pharmacological nature, the provision of health education, the encouragement of physical exercise, and the promotion of healthy eating, through motivation and support for people with CC in carrying out self-care tasks, which promotes a sense of security, reducing anxiety^(27)^.

A study conducted in Denmark, which began with an in-person interview with chronic patients, followed by TM, found that despite the challenges of introducing a new technology into daily care, it was able to contribute to the improvement of the health condition of this population. In addition, professionals were able to more effectively monitor vital signs, treatment adherence, and provide personalized guidance to the study participants^(20)^. Similarly, a study that used TM as a strategy to support care delivery and engage patients with HTN, DM, and heart failure in their home care showed moderate to high adherence to the strategy among all participants^(28)^.

In turn, a study that analyzed the effectiveness of using telephone support by nurses and home-based TM found these strategies viable for supporting the care of people with chronic diseases, as they improved survival rates and reduced costs compared to usual care provided^(29)^. It is worth noting that personalized guidance can help strengthen the bond between the professional and the service user, which leads to greater engagement of individuals with their health condition^(4)^.

The use of ICTs from the perspective of the CCM can bring benefits to healthcare systems by reducing costs, increasing care efficiency, and empowering users. It also serves as a strategy for empowering nurses in organizing the work process within the PHC context^(30)^. The importance of effective communication strategies was highlighted, even in a study that investigated the intervention components and contextual factors that most facilitate care transitions for complex elderly patients, using the Delphi technique with 23 experts. Among the top five ranked factors were: (a) educating and training patients, their families, and caregivers on self-management skills; (b) using standardized documentation tools and comprehensive communication strategies during care transitions^(31)^.

However, to fully achieve these benefits, it is essential to consider the training and involvement of the multidisciplinary team in PHC. Their participation in teaching-learning activities is necessary and relevant for future contributions to service improvements, enhancing care, and focusing on meeting the needs of the community. To promote the use of technologies by health professionals, especially in PHC, it is crucial to improve the quality of care provided to users with chronic conditions^(32)^. A study conducted in Pernambuco, which analyzed ICTs as a health education tool, found that professionals described health education as an aspect of care, with a horizontal characteristic and a holistic view, involving all individuals as participants in the process of building health^(33)^.

It is necessary to understand the functioning and use of ICTs available for TM, as some healthcare professionals are not sufficiently familiar with the practicality of applications available for smartphones and other communication devices that allow access to and monitoring of people with NCDs. Amid the technological advancements, the use of ICTs provided for in the National Policy for Permanent Health Education (PNEPS in Portuguese) enhances the dissemination of knowledge, facilitates learning, enables remote monitoring, overcomes geographical barriers, and improves access to health services^(34)^. Thus, awareness and educational actions regarding the use of ICTs among professionals, managers, and service users are essential for their future incorporation into the teams’ work processes. Moreover, discussions about the sustainable funding of this complementary strategy for monitoring people with NCDs can support its incorporation into PHC^(27)^.

It is worth noting that the use of technologies in the management of NCDs can strengthen the bond between professional and user and enable remote monitoring, which, in turn, allows for the clarification of doubts and the promotion of self-care, reducing the number of emergency visits and hospitalizations compared to traditional clinical management, and avoiding unnecessary medical tests ^(4)^. This experience benefits not only the users and the community served but also health professionals, who have the opportunity to experience a new approach that promotes and understands health beyond the biomedical perspectiv^(35)^.

However, the workload and high staff turnover mentioned in this study can hinder the use of new strategies, such as TM in monitoring people with CC. According to the CCM, one of the attributes of the FHS clinic is the continuity of care, which values the long-term relationship between healthcare professionals and users. The relationship between them has intrinsic value and precedes the actual content of care, especially in cases of chronic conditions^(4)^. However, research conducted in Rio de Janeiro found that part of the workload perceived by PHC nurses is related to “care pressure” arising from the high volume of spontaneous demand^(36)^. The absence of professionals in health teams, combined with the diversity of management and care activities performed by PHC nurses, can hinder the implementation of activities related to the continuity of CC care. Thus, it is necessary to reflect on the organization of the work processes of nurses in PHC and the prioritization of actions that align with the principles of professional practice.

In the appropriate context, TM can be used as a shared responsibility tool in healthcare. This is because it allows the service user to be more active in their treatment, regularly monitoring their health status and reporting any changes to the healthcare team. The use of support strategies for self-care, which include health status assessment, goal setting, care planning, problem-solving actions, and monitoring, are central elements of the CCM and contribute to greater user awareness of their condition and greater responsibility for their own health^(35)^.

Although the study participants reported some challenges in using TM in the routine of PHC services, two of them are definitely beyond their control: user adherence and lack of time. Therefore, it is essential that healthcare professionals are open to learning and adapting to this new approach to remote monitoring. They need to understand the benefits of TM and be willing to integrate it into their daily clinical practices. This may involve additional training and familiarization with the tools and platforms to be used^(37)^. TM is highlighted in the literature as a way to reduce medical expenses and medication costs^(38)^. These costs have an economic impact on family income, especially for the elderly, who have few social and community support systems.

When used as a tool for managing NCDs, TM enhances the strategies for controlling these diseases, reducing the use of healthcare services, which results in lower costs for the State^(39)^. However, there are challenges in implementing and using TM as a care monitoring technology. There is a need to work on acceptance by both professionals and users, which is hindered by socioeconomic and psychological factors in the population, such as difficulty interacting with technology, privacy concerns, and preferences for in-person consultations^(36)^, as pointed out by the participants in this study.

A study conducted with elderly individuals identified challenges to be faced (by both service users and professionals) for integrating ICT interventions into the work process, such as knowledge gaps, lack of willingness to adopt new skills, reluctance to use technologies, slow Internet connectivity, and the lack of an adequate reimbursement policy. However, the positive perspective of users regarding the use of ICTs overcame these challenges, as it contributed to increased motivation, support for self-management actions, a sense of security, and reduced anxiety^(27)^.

The challenges faced in PHC are longstanding and involve the inadequate condition of the physical network of UBS, insufficient funding for infrastructure and investment in technologies, and a low number and diversity of human resources. These challenges have made it difficult to incorporate a humanized care model, as recommended by the CCM^(40)^. These challenges were also frequently cited by the professionals in the study and limited the incorporation of telemonitoring for promoting self-care and improving interaction with users with chronic conditions.

### Study limitations

A notable limitation of the study is that the participants were nurses who had previously attended awareness workshops on the use of TM. This circumstance may have significantly influenced their perceptions and opinions on the subject, as having received prior information and specific guidance on TM could have led to a positive predisposition toward the strategy.

### Contributions to the Field of Nursing, Health, or Public Policy

The study provides valuable insights into nurses’perceptions of TM in PHC and highlights the importance of human and structural resources for effective implementation. This information can be useful in planning and guiding actions aimed at improving care for people with chronic conditions in PHC.

## FINAL CONSIDERATIONS

The nurses showed interest in using the TM strategy, especially those who recognized its importance for frequent and high-quality monitoring. They considered that TM helps maintain contact/bond with users with chronic conditions and/or their families, facilitates regular follow-up, and provides important guidance, in addition to promoting effective communication, as recommended by the CCM. Furthermore, it reduces the need for unnecessary travel, without disregarding in-person consultations. However, they noted the need to provide human and technological resources, as well as reorganize work processes to make the implementation of TM in PHC effectively feasible.

Based on the findings, it is concluded that investments in infrastructure, information technology, and human resources are necessary, along with ongoing actions to raise awareness among professionals about establishing care using TM. Additionally, the strategy should involve multidisciplinary teams, encouraging each healthcare worker’s interest and participation in the treatment, monitoring, and evaluation of users, as well as ensuring the strengthening of primary care, assisting in the process of caring for these individuals.
