# Supplementary material for: Nurses’ perspectives on the use of telemonitoring in the management of people with diabetes and hypertension
Source: Rev Bras Enferm. 2025 Jan 13;77(6):e20230481. doi: 10.1590/0034-7167-2023-0481 (PMC11731851; doi:10.1590/0034-7167-2023-0481)
Supplement: Translation

# Translation: Perspectiva de enfermeiros sobre o uso do telemonitoramento no acompanhamento de pessoas com diabetes e hipertensão

**DOI:** 10.1590/0034-7167-2023-0481pt

**Authors:** Camila Wohlenberg Camparoto, Maria do Carmo Fernandez Lourenço Haddad, Elen Ferraz Teston, Pamela dos Reis, Sonia Silva Marcon

**Affiliations:** I Universidade Estadual de Maringá. Maringá, Paraná, Brasil; II Universidade Estadual de Londrina. Londrina, Paraná, Brasil; III Universidade Federal do Mato Grosso do Sul. Campo Grande, Mato Grosso do Sul, Brasil; IV Universidade do Vale do Itajaí. Itajaí, Santa Catarina, Brasil

**Keywords:** Telemonitoramento, Doença Crônica, Atenção Primária à Saúde, Enfermagem, Cuidado

## Abstract

**Objetivos:**

compreender a perspectiva de enfermeiros sobre o uso do telemonitoramento no acompanhamento de pessoas com diabetes *mellitus* tipo 2 e hipertensão arterial na atenção primária.

**Métodos:**

pesquisa qualitativa com dezesseis enfermeiros de oito municípios do Paraná. Os dados foram coletados entre novembro de 2022 e janeiro de 2023, mediante entrevistas presenciais ou remotas, audiogravadas, previamente agendadas, e submetidas à análise de conteúdo.

**Resultados:**

para os enfermeiros, o telemonitoramento favorece o conhecimento do usuário acerca dessas condições, a comunicação e o vínculo com a equipe, e sua produtividade; porém, inexistência de recursos eletrônicos e equipamentos, rotatividade da equipe, baixa adesão dos usuários e indisponibilidade de tempo dos profissionais constituem desafios importantes.

**Considerações Finais:**

implementação e operacionalização eficaz do telemonitoramento no acompanhamento de pessoas com diabetes e hipertensão envolvem potencialidades e barreiras, sendo imprescindível a disponibilidade de recursos humanos e tecnológicos, apoio da gestão e disposição de profissionais e usuários.

## INTRODUÇÃO

A transição epidemiológica, nutricional, demográfica e tecnológica tem desencadeado o aumento das Doenças Crônicas Não Transmissíveis (DCNT), resultando em custos elevados para os sistemas de saúde e impactos negativos na qualidade de vida. Frente a isso, torna-se premente a necessidade de encontrar e valorizar estratégias custo-efetivas e aplicáveis ao contexto da atenção primária, com enfoque na prevenção e manejo dessas doenças^(1)^.

Segundo a Organização Mundial da Saúde (OMS), as Doenças Crônicas (DC) são responsáveis por 41 milhões de mortes todos os anos. Elas constituem um problema global, visto que apenas 25% das pessoas com DC são assistidas por serviços de saúde, e metade delas alcança as metas clínicas de cuidado pretendidas. Esse resultado se deve, em grande parte, ao acesso inadequado aos cuidados de saúde e à dificuldade na autogestão da própria condição de saúde^(2)^.

Embora o propósito do Sistema Único de Saúde (SUS), desde a sua criação, seja a reorganização do modelo assistencial do país, ainda persistem grandes desafios, sobretudo em relação ao manejo adequado das condições crônicas e ao engajamento do indivíduo nas ações de autocuidado. Essa situação desafia os pesquisadores, os próprios serviços e os profissionais de saúde a buscarem, constantemente, modelos assistenciais que possam proporcionar melhores resultados^(3)^.

Considerando essa perspectiva, foi proposto em 2011 o Modelo de Atenção às Condições Crônicas (MACC), na busca de uma atenção contínua e proativa no SUS^(4)^, tendo como propósito contribuir para a redução do agravamento e da mortalidade por condições que são sensíveis à Atenção Primária em Saúde (APS), como as doenças crônicas^(5)^. A APS constitui a principal porta de entrada no sistema de saúde e tem como prioridade a prevenção das doenças e a promoção da saúde, sem renunciar aos serviços assistenciais^(6)^.

Por sua vez, o MACC é um modelo assistencial que, para ser efetivamente incorporado, demanda capacitação profissional, pois envolve mudanças no processo assistencial às pessoas com condições crônicas^(7)^. Investir na formação em serviço permite a atuação adequada dos profissionais e gestores de saúde que atuam no âmbito da APS, sobretudo na atenção dispensada pelas equipes da Estratégia Saúde da Família (ESF). Uma assistência adequada pode ajudar a reduzir as hospitalizações e os óbitos derivados do agravamento das condições crônicas^(8)^.

Algumas estratégias têm se mostrado exitosas na prevenção e manejo da Hipertensão Arterial (HA) e do Diabetes *Mellitus* (DM), especialmente o tipo 2 (DM2), cujo controle está muito relacionado à mudança de comportamento e estilo de vida. Dentre essas estão incluídos os protocolos assistenciais, o trabalho em equipe — com comunicação adequada entre seus integrantes —, o seguimento e monitoramento das condições crônicas, atividades de promoção da saúde, a identificação de casos novos e de pessoas com maior risco, entre outras^(8)^. O desafio, no entanto, está no gerenciamento e manutenção dessas ações^(9)^.

O monitoramento no setor saúde ganhou relevância durante a pandemia da Covid-19, visto que, com a crise sanitária instalada, marcada pela falta de leitos hospitalares e isolamento social, os casos notificados precisaram ser monitorados^(10)^. Nesse cenário, as pessoas acometidas por condições crônicas foram consideradas muito vulneráveis e, por isso, orientadas a evitar os serviços de saúde, ao mesmo tempo em que precisavam ter sua condição clínica minimamente acompanhada. Deste modo, percebeu-se que boa parte dessas pessoas, desde que de forma programada, poderia ser acompanhada à distância, por telemonitoramento (TM), sem prejuízos para sua saúde.

Cabe destacar que, na APS, o TM consiste na utilização de meios que permitem ao profissional de saúde acompanhar os parâmetros clínicos e obter informações relevantes do processo saúde-doença dos usuários, seja por meio de aplicativos de smartphones ou outras plataformas, correios eletrônicos (e-mail e SMS) ou ligações telefônicas^(11)^. O acesso a essas tecnologias e recursos facilita o acompanhamento da evolução e eficácia do tratamento de forma remota pela equipe de saúde e por parte do usuário e/ou familiares^(12)^.

Antes mesmo da pandemia, com base em resultados de estudos realizados em outros países, o TM de condições crônicas já estava sendo discutido no Brasil, sendo ressaltada a possibilidade de redução de gastos, além de outros benefícios, como redução nos atendimentos nas salas de emergência, internações agudas, reinternações e tempo de cuidados domiciliares, entre outros^(13)^. Inclusive, algumas operadoras de saúde suplementar já colocavam em prática essas ações, considerando os resultados positivos para o usuário^(14,15)^ e para a instituição^(16)^ relatados na literatura.

Contudo, no Brasil, estudos sobre o uso dessa tecnologia assistencial ainda são incipientes e isolados, mas já foi utilizado para avaliação dos efeitos no controle da obesidade, sendo que um deles destacou a facilidade de acesso dos usuários aos profissionais de saúde^(17)^, e o outro constatou a diminuição do risco para a síndrome metabólica^(18)^. No âmbito internacional, estudos atestam os benefícios para pessoas com diferentes condições crônicas^(11,19)^. Pesquisa realizada na Dinamarca, por exemplo, constatou que, na perspectiva de enfermeiros, médicos e pacientes com doenças crônicas, apesar de ter sido um desafio inserir uma tecnologia como o TM no cotidiano do cuidado, ela foi capaz de colaborar com a melhora da condição de saúde dos pacientes^(20)^.

Por sua vez, um estudo de revisão de ensaios clínicos randomizados abordando intervenções voltadas para a autogestão de doenças crônicas apontou que o TM, dentre outras estratégias, pode beneficiar a mudança e sustentar esforços de longo prazo para conter a progressão de doenças e incentivar comportamentos de saúde, melhorando a qualidade de vida do usuário e reduzindo o uso de serviços de saúde^(21)^. Intervenções educativas remotas também demonstraram influência positiva no letramento em saúde e no conhecimento sobre hipertensão e até mesmo no estilo de vida. Esses aspectos destacam o potencial do TM como forma de acompanhamento e promoção de saúde, favorecendo a gestão da doença^(22)^. Contudo, um estudo que investigou a disposição de enfermeiros da APS em utilizar o TM como recurso auxiliar no acompanhamento de pessoas com DM e/ou HA constatou que ela era menor entre os mais velhos, com maior tempo de formação e de atuação na APS, e que não acreditavam que essa estratégia assistencial facilita a comunicação com o paciente^(23)^.

Nesse sentido, questiona-se: a utilização do TM como estratégia auxiliar é viável para acompanhar as pessoas com condições crônicas na APS?

## OBJETIVOS

Compreender a perspectiva de enfermeiros(as) sobre o uso do TM no acompanhamento de pessoas com diabetes *mellitus* tipo 2 e hipertensão arterial na APS.

## MÉTODOS

### Aspectos éticos

No desenvolvimento do estudo, foram seguidas todas as diretrizes éticas estabelecidas pelas Resoluções 466/2012 e 510/2016, do Conselho Nacional de Saúde, e as Diretrizes para Procedimentos de Pesquisa em Qualquer Fase em Ambientes Virtuais – CONEP/2021. O projeto foi aprovado pelo Comitê de Ética em Pesquisa com Seres Humanos da instituição signatária. Os participantes manifestaram concordância em participar do estudo assinando o Termo de Consentimento Livre e Esclarecido (TCLE). Nos casos de entrevistas remotas, o TCLE foi enviado via formulário online. Para garantir o anonimato na apresentação dos resultados, os trechos dos relatos estão identificados com a letra E, para designar entrevistado, e um número indicativo da ordem de realização da entrevista, exemplo: E1.

### Referencial teórico-metodológico

Foi utilizado como base conceitual o MACC, que teve sua estruturação fundamentada nos princípios do SUS. Ele propõe a organização das atividades de assistência à saúde da população a partir de cinco níveis de intervenção, de acordo com os determinantes e necessidades da população^(4)^.

### Tipo de estudo

Trata-se de um estudo descritivo exploratório, de natureza qualitativa, que não tem como propósito obter resultados generalizáveis. O *consolidated criteria for reporting qualitative research* (COREQ) foi utilizado para guiar o relatório da pesquisa^(24)^.

### Procedimentos metodológicos

Os informantes do estudo foram os enfermeiros que atuam na APS de municípios que integram a 15ª Regional de Saúde do Paraná e que participaram de oficinas de sensibilização para o uso do TM no acompanhamento de pessoas com condições crônicas. Pelo menos três tentativas de contato com os participantes das oficinas foram realizadas com o intuito de convidá-los a participar do estudo e agendar as entrevistas, em caso de aceite. Todos os que aceitaram foram incluídos.

### Cenário de estudo

O cenário do estudo abrangeu os municípios que integram a 15ª Regional de Saúde (RS) do Paraná. Todos os enfermeiros atuantes na APS dos 30 municípios dessa RS foram convidados a participar de uma intervenção, mas apenas 16, pertencentes a oito municípios, participaram efetivamente. A intervenção proposta envolveu leituras prévias, atividades práticas — como a testagem dos instrumentos elaborados — e cinco oficinas de sensibilização sobre o uso do TM no acompanhamento de pessoas com condições crônicas. As oficinas tiveram duração de 60 a 90 minutos e foram realizadas semanalmente, no período de maio a junho de 2020, de forma remota, em dia e horário estabelecidos pelos participantes como mais convenientes.

No encontro anterior ao início da intervenção, os enfermeiros foram convidados a relatar e refletir sobre as características e os resultados dos atendimentos às pessoas com HAS e/ou DM em seus municípios. O grupo concluiu que o atendimento era fragmentado e focado nas queixas/condições apresentadas pelos usuários, com base nas recomendações contidas na Linha Guia do Estado do Paraná^(25)^.

Após o compartilhamento de experiências e discussões sobre possibilidades assistenciais orientadas por diversas leituras, o grupo elaborou, de forma participativa, um Protocolo de TM (ligação telefônica e mensagens de WhatsApp) para acompanhamento de pessoas com HA e/ou DM na APS, além de alguns instrumentos para direcionar a assistência e seu registro. O protocolo elaborado inclui as seguintes etapas: 1) Identificar usuários aptos para participar; 2) Determinar o grau de risco do usuário; 3) Realizar consulta de enfermagem presencial para identificação de problemas, definição de metas a serem alcançadas e da periodicidade das ligações telefônicas; 4) Elaborar plano de assistência; 5) Realizar os telecontatos para acompanhar o andamento do plano de cuidados; e 6) Avaliar o alcance das metas.

Por fim, o grupo definiu que o protocolo deveria ser testado e, para isso, estabeleceu que cada enfermeiro deveria implementar o TM pelo período de 12 meses junto a 30 usuários cadastrados em sua Unidade Básica de Saúde (UBS).

### Coleta de dados

Os dados foram coletados no período de novembro de 2022 a janeiro de 2023, 17 meses após as oficinas, por meio de entrevistas semiestruturadas, audiogravadas após consentimento, realizadas de modo remoto ou presencial, de acordo com a escolha do participante, em dia, horário e local de sua preferência.

As entrevistas tiveram duração média de 18 minutos e foram guiadas por um roteiro semiestruturado, composto por duas partes: a primeira com questões destinadas à caracterização sociodemográfica dos(as) participantes, e a segunda composta pela questão norteadora: “Qual a sua opinião sobre o TM hoje?”. Algumas questões de apoio foram utilizadas, como:“Você conseguiu implementar? Fale mais sobre isso”;“Como foi sua experiência com o TM?”; e “Quais foram suas dificuldades?”.

Todas as entrevistas foram realizadas pela mesma pesquisadora (enfermeira, mestranda em enfermagem, com alguma experiência na coleta e análise de dados qualitativos por ter participado de outras pesquisas), que não possuía nenhuma relação com os(as) participantes do estudo.

### Análise dos dados

Para o tratamento dos dados, as entrevistas foram transcritas na íntegra e submetidas à análise de conteúdo, na modalidade temática, que segue três etapas^(26)^. Na etapa de pré-análise, realizou-se a organização do material transcrito e a leitura repetitiva, com identificação das unidades de registro. Na etapa de exploração do material, foi feita a codificação e identificação das categorias provisórias, cujos termos foram derivados da leitura dos dados. Por fim, na etapa de tratamento e interpretação dos dados, foi realizado o reagrupamento dos códigos por semelhança, com confirmação das duas categorias provisórias, seguido de inferência sobre suas relações e interpretação à luz do MACC.

## RESULTADOS

Dos 16 enfermeiros participantes do estudo, apenas um era do sexo masculino. Todos tinham tempo de atuação na APS superior a cinco anos (média de 15 anos), sendo que dez possuíam especialização e duas, mestrado. Da análise dos dados, emergiram duas categorias, que serão apresentadas a seguir.

### Potencialidades do telemonitoramento no acompanhamento de usuários com hipertensão e diabetes

Dentre os benefícios da utilização do TM, os enfermeiros destacaram a possibilidade de transmitir aos usuários mais informações relativas à sua condição de saúde, o que eles acreditam qualificar a assistência.

*Eles tiram bastante dúvidas, eles acabam interagindo mais [...] e a gente já passa as orientações, daí eles dão o feedback, fazem o que é orientado.* (E5)

*A maioria dos usuários com doenças crônicas tem um grau de conhecimento baixo* [...]*, às vezes [...] não sabem o que pode comer, os riscos que tem de uma [...] lesão pequena, que aquela lesão pode demorar para cicatrizar*[...]*e acaba desenvolvendo problemas mais graves, que precisamdemaisrecursos...deumgraumaiordeatendimento*[...]*com maiscustos*[...].*Facilitaocontato*[...]*comisso,achoqueaqualidadede vida deles melhora muito, muito mesmo.* (E10)

O contato remoto facilita a comunicação e o acesso a informações.

*Aquelas pessoas mais jovens que trabalham, tem o WhatsApp, facilita bastante* [o contato]. (E2)

*Não precisar se deslocar até a unidade e ao mesmo tempo fazer um reforço, para ele estar lembrando como tem que fazer* [...]. (E11).

Para além, reconhecem a importância desse recurso como estratégia para estabelecer, e até mesmo fortalecer o vínculo com os usuários, sobretudo aqueles que percebem os telecontatos como uma forma de assistência mais individualizada e que lhes permite se sentirem mais valorizados.

*É uma forma de ter um contato mais rápido e mais frequente, contínuo com o paciente* [...]*, com a comunidade [...] os pacientes recebem muito bem, eles até gostam dessa atenção mais individualizada [...] como se ele tivesse mais valorizado.* (E5)

*É a possibilidade de vínculo, você já vai formando na primeira consulta. E depois, você mantém esse vínculo com os pacientes pelo monitoramento, alguns se sentem valorizados por terem um profissional da saúde ligando* [...]*, dando orientações e perguntando como você está* [...]. (E9)

Destacaram que o TM também traz benefícios para o serviço, pois o torna mais produtivo, ao possibilitar atendimento a um número maior de usuários no mesmo espaço de tempo.

*No tempo que você gastaria em visitar cinco usuários, você consegue, pelo TM, atender dez usuários.* (E10)

Também colocaram em relevo o importante papel do TM durante o período pandêmico, devido à dificuldade de locomoção dos pacientes até o serviço de saúde.

*Acho que ele veio muito a calhar em relação ao período pandêmico [...] foi possível acompanhar os pacientes que mais se afastaram das unidades* [...]. *Então [...] o TM [...] permitiu com que a gente ficasse um pouco mais próximo desses usuários* [...]. (E10)

*Na pandemia [...] os pacientes não vinham até a unidade e era uma forma da gente se comunicar, saber se estavam bem, se estavam tomando a medicação, saber como a pressão arterial ou a glicemia, daqueles que tinham aparelho em casa, estava ficando, se estavam controlando* [...]. (E13)

*Conversar com o paciente e saber um pouco mais do cotidiano dele [...] em relação aos hábitos alimentares, dava para perceber muita coisa naquelas consultas. Conheci mais meus pacientes, mesmo aqueles que eu já tinha convívio diário na UBS* [...]. (E11)

Os relatos incluídos nesta categoria destacam as potencialidades do TM na perspectiva das enfermeiras, com ênfase na transmissão de conhecimento sobre a doença e seu tratamento, facilidade de comunicação, favorecimento de vínculo e qualificação da assistência e produtividade da equipe.

### Adversidadesencontradasnarealizaçãodotelemonitoramento

Os enfermeiros relataram que uma das barreiras que enfrentaram na implementação do TM foi a indisponibilidade de equipamentos específicos, a exemplo de linhas telefônicas ou aparelhos móveis.

*Tive que usar o celular particular [...] e isso acaba atrapalhando* [...]. *Para gente utilizar o fixo, tinha que usar uma linha e são poucas linhas.* (E10)

*A gente tinha dificuldade com ligação, porque só tem uma linha telefônica e aqui são duas equipes, então dificultava nisso.* (E12)

*Em questão de telefone [...] se usar o próprio, a gente consegue dinamizar. Depender do telefone da UBS, eu acho um pouco mais complicado,* [...]. *É uma barreira.* (E4)

*Só tem um mês e meio que eles colocaram o WhatsApp, porque antes disso não tinha [...] essa parte tecnológica acho que ajuda.* (E2)

*Uma questão que a gente não conseguiu, foi o celular [...] porque o município não assumiu o compromisso de comprar.* (E10)

Isso porque os aplicativos de mensagens (*WhatsApp*) foram apontados como primeira opção para a efetivação do contato com os usuários.

*A gente não tem WhatsApp, que era uma possibilidade de estar conversandocomopaciente*[...]. *Entãoésóotelefonefixomesmo.*(E6)

*A gente não tinha WhatsApp, então o pouco que conseguíamos falar era utilizando o celular das ACS* [Agentes Comunitárias de Saúde] *e isso dificultava muito, porque não era específico para o TM, era WhatsApp geral* [...]. (E3)

Contudo, segundo os enfermeiros, alguns usuários demonstravam insatisfação com o atendimento quando os contatos eram feitos por aplicativos, ou então não respondiam as mensagens.

*As orientações só por WhatsApp, eles não estavam satisfeitos*

[...] *queriam vir, embora a gente fizesse o mesmo reforço* [...]. (E8)

Discorreram também sobre barreiras relacionadas ao espaço físico para a realização da consulta presencial, que antecede o TM.

*A dificuldade é que o espaço físico estava sendo limitado [...] não temos estrutura, nós começamos a ter [...] certo impasse.* (E9)

*Eu não tenho um consultório específico de enfermagem [...] que eu poderia ficar à vontade para fazer isso. Então geralmente o atendimento é o auxiliar e o enfermeiro juntos [...] para mim é bem [...] complicado. A estrutura da nossa unidade não comporta [...] tantas pessoas, tantos atendimentos que a gente faz.* (E16)

O fato de a equipe estar incompleta também foi apontado como fator desencadeador de sobrecargas, o que comprometeu a implementação do TM.

*Nós estamos sem um enfermeiro [...] acho que o principal problema é a falta de profissionais [...] é difícil porque a gente sabe que é humanamente impossível* [...]. (E12)

*Nós ficamos sem médico na unidade, tivemos muitas complicações [...] e a gente não conseguiu realizar o programa como queríamos* [...]. (E14)

Até mesmo as UBS que dispunham dos recursos tecnológicos e estruturais necessários enfrentaram dificuldades para implementar o TM. Uma das dificuldades referidas estava relacionada à necessidade de o usuário se deslocar até a UBS sem uma queixa específica e ao tempo despendido para realizar a consulta inicial de enfermagem.

*Eu acho que a maior dificuldade foi a captação e disponibilidade para irem até a UBS para a consulta de enfermagem dos pacientes.* (E11)

*Às vezes a dificuldade de o paciente estar vindo [...] porque moram um pouco longe, ou porque tem dificuldade de andar e não tem, um [...] um meio de locomoção, um carro, alguém que traga* [...]. (E1)

*Foi complicado para cadastrar, porque é uma consulta mais demorada, que a gente tem que avaliar o paciente e eles querem sair ou fugir as vezes, da consulta.* (E2)

Outro aspecto citado, foi o fato de os usuários não aderirem aos propósitos desse modelo de assistência, voltado para o autocuidado e monitoramento contínuo por parte do profissional.

*Aquelas pacientes que realmente precisavam, eles aderiram, mas aqueles que estava mais controlado, eles já não [...] parece que eles não se importaram tanto.* (E2)

Algumas falas indicaram que parte dos usuários apresentou resistência às mudanças no modelo de assistência.

*A resistência maior é do paciente em mudar o modelo de assistência, porque enquanto tudo estava parado, por conta da pandemia eles aceitaram, agora como começou a equilibrar a vacinação, perderam o medo de vir na UBS, então eles preferem vir ao consultório [...] do que via telefone.* (E2)

*O pessoal tem bastante dificuldade ainda, dificuldades de se expressar mesmo, o que não substitui uma conversa presencial são os próprios pacientes [...] a dificuldade em expressar o que sentem [...] o que está acontecendo, é a dificuldade com o meio de comunicação.* (E3)

A limitação que alguns usuários apresentavam em relação à utilização das tecnologias foi outra barreira mencionada.

*O que não favoreceu é [...] que não tinham tanto domínio com*

[...] *a tecnologia.* (E13)

*A questão de orientação [...] acho que às vezes você está no telefone orientando [...] principalmente o idoso, ele se confunde muito pelo telefone.* (E4)

*Alguns tem dificuldades até mesmo de entender, a gente fala uma coisa e entendem outra* [...]. (E12)

Assim como o fato de os usuários não atenderem os chamados telefônicos ou nem mesmo responderem as mensagens escritas no *WhatsApp*.

*A maioria não atendeu, eu até formulei e coloquei no WhatsApp [...] me identifiquei [...] mas muitos não responderam [...] e a maioria me respondia assim meio [...] evasivo.* (E2)

*Não atende à ligação, não responde, vê a mensagem, visualiza, mas não quer responder* [...]. (E9)

*Os pacientes às vezes não atendem o telefone, você tenta uma, tenta duas, tenta três … e eles não atendem* [...]. (E14)

Relataram ainda a relevância do vínculo dos profissionais com os usuários para a adesão, mas também as dificuldades encontradas em criar e manter esse vínculo frente a alta rotatividade nos serviços.

*Os pacientes que tinham um vínculo maior, eles abraçaram um pouco mais a ideia. Se ele tem um vínculo maior com o profissional a facilidade de adesão é melhor* [...]. (E2)

*A rotatividade de profissionais acabou quebrando todo o vínculo que no início começou, e, querendo ou não o próprio usuário acaba se afastando, não quer participar porque ele não tem vínculo.* (E12)

Por último, destacaram a sobrecarga de atividades decorrente, sobretudo, da realização da Consulta de Enfermagem, que embora seja uma atribuição do enfermeiro e umas das ações prioritárias no contexto da APS não era realizada rotineiramente nos serviços em estudo.

*A consulta em si para o enfermeiro fazer acho que é bem complicado [...]. A gente já tem muita coisa no dia a dia.* (E4)

*A questão da consulta de enfermagem, do exame físico em si, a gente é cobrado, mas, no momento não é feito, não tem como* [...]. (E6)

*Se eu falar que tive tempo suficiente, não tive* [...]. *No começo foi mais difícil, [...] demandou muito tempo [...] para aquele preenchimento de ficha. A gente não estava fazendo completo. Eu acho que o maior problema foi a falta de tempo* [...]. (E8)

*É difícil de fazer, porque as equipes estão muito sobrecarregadas, com muita coisa a fazer, muitas demandas, pós-pandemia, muita coisa para organizar, reorganizar* [...]. (E9)

Os relatos apresentados nessa categoria mostram que os profissionais têm interesse em realizar o TM junto aos usuários cadastrados em sua equipe, mas encontram diversas dificuldades relacionadas à estrutura física, recursos eletrônicos limitados, rotatividade dos membros da equipe, adesão dos usuários e escassez de tempo para realização das atividades relacionadas.

## DISCUSSÃO

Esse estudo permitiu compreender que os enfermeiros percebem potencialidades na utilização do TM como estratégia auxiliar para acompanhar as pessoas com DC na APS, e os participantes demonstraram interesse em utilizá-la. Contudo, ressaltaram que a capacidade para realizar mudanças na assistência, a partir da utilização de novas tecnologias, relaciona-se com aspectos básicos, como a disponibilidade de equipamentos eletrônicos de comunicação, de estrutura física e de recursos humanos.

Condições crônicas como HA e DM são acompanhadas pelas equipes atuantes na APS, responsáveis por oferecer cuidados voltados à promoção da saúde e prevenção de doenças de forma contínua, abrangente e coordenada. Contudo, os enfermeiros do presente estudo apontaram que o manejo é embasado em um modelo de atendimento biomédico e curativista, o que tem sido destacado como uma barreira para a oferta de uma assistência que insira o usuário na tomada de decisão sobre sua condição de saúde para diminuir as complicações^(3)^, conforme proposto pelo MACC.

Nesse sentido, consideraram positiva a possibilidade de utilizarem uma estratégia para acompanhar os usuários da APS de forma individualizada. Esse fato corrobora o resultado de estudo realizado em Santa Catarina^(12)^, que, embora tenha identificado a necessidade de um melhor planejamento com acompanhamento mais regular e o estabelecimento de metas específicas no plano de cuidados, mostrou que o monitoramento de usuários com HA e DM, especialmente aqueles insulinodependentes, teve um impacto positivo na redução de complicações, evitando hospitalizações e consultas desnecessárias. Os enfermeiros, portanto, identificaram que a abordagem individualizada aos usuários foi benéfica^(12)^.

Na área da saúde, as Tecnologias de Informação e Comunicação (TICs) são utilizadas para ajudar as pessoas a autogerirem doenças crônicas e demonstram efeitos positivos^(27)^. As vantagens da utilização de intervenções com TICs incluem sua natureza não farmacológica, a oferta de educação em saúde, o incentivo ao exercício físico e a promoção de uma alimentação saudável, por meio da motivação e apoio às pessoas com DC na realização de tarefas de autocuidado, o que promove uma sensação de segurança, reduzindo a ansiedade^(27)^.

Estudo realizado na Dinamarca, que teve início com uma entrevista presencial com doentes crônicos, seguida pelo TM, verificou que, apesar dos desafios de inserir uma nova tecnologia no cotidiano do cuidado, essa foi capaz de contribuir para a melhoria da condição de saúde dessa população. Além disso, os profissionais puderam monitorar de forma mais eficaz os sinais vitais, a adesão ao tratamento e fornecer orientações personalizadas aos participantes do estudo^(20)^. Do mesmo modo, estudo que utilizou o TM como estratégia para apoiar a prestação de cuidados e envolver os pacientes com HA, DM e insuficiência cardíaca em seus cuidados no domicílio apontou nível de adesão à estratégia de moderada a alta de todos os participantes^(28)^.

Por sua vez, estudo que analisou a efetividade da utilização do suporte telefônico pelo enfermeiro e do TM domiciliar apontou essas estratégias como viáveis para apoiar as ações de cuidado de pessoas com doença crônica, por melhorarem a sobrevida e o custo quando comparadas com os cuidados habituais ofertados^(29)^. Cabe destacar que as orientações personalizadas podem favorecer o fortalecimento do vínculo entre o profissional e o usuário do serviço, o que proporciona maior engajamento do indivíduo com sua condição de saúde^(4)^.

A utilização de TICs na perspectiva do MACC pode trazer benefícios para os sistemas de saúde, reduzindo os custos, aumentando a eficiência do cuidado e o empoderamento dos usuários, além de constituir uma estratégia de empoderamento do enfermeiro em relação à organização do processo de trabalho no contexto da APS^(30)^. A importância de estratégias eficazes de comunicação foi destacada, inclusive, em um estudo que investigou os componentes de intervenção e os fatores contextuais que mais facilitam as transições de cuidados para idosos complexos, utilizando a técnica Delphi com 23 especialistas. Dentre os cinco melhores classificados, tem-se: (a) educar e treinar pacientes, seus familiares e cuidadores sobre habilidades de autogestão; (b) utilizar ferramentas de documentação padronizadas e estratégias de comunicação abrangentes durante as transições de cuidados^(31)^.

No entanto, para que esses benefícios sejam plenamente alcançados, torna-se premente considerar a capacitação e o envolvimento da equipe multiprofissional na APS. Sua participação nas atividades de ensino-aprendizagem se faz necessária e relevante para futuras contribuições nas melhorias dos serviços, aprimorando o cuidado e tendo o olhar voltado para o atendimento das necessidades da comunidade. Para promover a utilização de tecnologias por profissionais de saúde, sobretudo na APS, é preciso aumentar a qualidade do cuidado que é oferecido aos usuários com condições crônicas^(32)^. Estudo realizado em Pernambuco, que analisou as TICs como ferramenta de educação em saúde, observou que os profissionais descreveram a educação em saúde como uma face do cuidado, com uma característica horizontal e uma visão holística, colocando todos os indivíduos como participantes do processo de construção da saúde^(33)^.

É necessário conhecer o funcionamento e o emprego das TICs disponíveis para o TM, pois alguns profissionais da saúde não conhecem suficientemente a praticidade dos aplicativos disponíveis para smartphones e outros equipamentos de comunicação, que permitem o acesso e o acompanhamento de pessoas com DCNT. Em meio ao advento tecnológico, o uso das TICs previstas na Política Nacional de Educação Permanente em Saúde (PNEPS) potencializa a socialização do conhecimento, facilita a aprendizagem, permite o acompanhamento à distância, superando barreiras geográficas e facilitando o acesso aos serviços de saúde^(34)^. Desse modo, ações de sensibilização e de educação sobre a utilização de TICs entre os profissionais, gestores e usuários do serviço são prementes para que possam futuramente ser incorporadas no processo de trabalho das equipes. Ademais, discussões sobre o financiamento sustentável dessa estratégia de complementação do acompanhamento das pessoas com DC podem favorecer sua incorporação na APS^(27)^.

Cabe salientar que o uso de tecnologias no manejo das DCNT pode promover o vínculo entre profissional e usuário e possibilitar o monitoramento remoto, o qual, por sua vez, permite o esclarecimento de dúvidas e a promoção do autocuidado, reduzindo o número de atendimentos de emergência e internações em comparação ao manejo clínico tradicional, evitando exames médicos desnecessários^(4)^. Essa vivência beneficia não apenas os usuários e a comunidade assistida, mas também os profissionais de saúde, que têm a oportunidade de experimentar uma nova modalidade que promove e compreende a saúde para além da visão biomédica^(35)^.

Entretanto, a sobrecarga e a alta rotatividade profissional citadas neste estudo podem prejudicar a utilização de novas estratégias, como o uso do TM no acompanhamento das pessoas com DC. Segundo o MACC, a clínica da ESF tem como um de seus atributos a longitudinalidade do cuidado, que valoriza a relação de longo prazo entre profissional de saúde e usuários. O relacionamento entre ambos tem um valor em si e antecede o próprio conteúdo do cuidado, especialmente nos casos das condições crônicas^(4)^. Contudo, pesquisa realizada no Rio de Janeiro verificou que parte da sobrecarga de trabalho percebida pelos enfermeiros da APS está relacionada à ocorrência da“pressão assistencial”, oriunda do elevado volume de atendimentos por demanda espontânea^(36)^. A ausência de profissionais nas equipes de saúde, somada à diversidade de atividades de gerência e cuidado realizadas pelos enfermeiros na APS, pode prejudicar a implementação de atividades relacionadas à continuidade do cuidado das DC. Assim, torna-se necessário refletir sobre a organização do processo de trabalho do enfermeiro na APS e sobre a priorização de ações que convergem com as premissas do exercício profissional.

No contexto adequado, o TM pode ser utilizado como um recurso de corresponsabilização no cuidado à saúde. Isso ocorre porque permite que o usuário do serviço seja mais ativo em seu próprio tratamento, monitorando regularmente seu estado de saúde e reportando quaisquer mudanças à equipe de saúde. O uso de estratégias de apoio para o autocuidado que incluam a avaliação do estado de saúde, a fixação de metas a serem alcançadas, a elaboração dos planos de cuidado, as ações de resolução de problemas e o monitoramento são elementos centrais no MACC e contribuem para maior conscientização do usuário sobre sua condição e maior responsabilidade sobre sua própria saúde^(35)^.

Embora os participantes do estudo tenham referido alguns desafios para a utilização do TM na rotina do serviço da APS, dois deles definitivamente não são de sua governabilidade: a adesão dos usuários e a escassez de tempo. Para tanto, é essencial que os profissionais de saúde estejam abertos a aprender e se adaptar a essa nova abordagem de monitoramento remoto. Eles precisam compreender os benefícios do TM e estar dispostos a integrá-lo em suas práticas clínicas diárias. Isso pode envolver treinamento adicional e familiarização com as ferramentas e plataformas a serem utilizadas^(37)^. O TM é destacado na literatura como um redutor de despesas médicas e de custos com medicamentos^(38)^. Esses custos impactam economicamente na renda das famílias, principalmente de idosos que contam com poucos dispositivos de apoio social e comunitário.

Quando usado como uma ferramenta de controle das DCNT, o TM valoriza as estratégias de controle dessas doenças, diminuindo o uso dos serviços de saúde, o que resulta em diminuição dos custos para o Estado^(39)^. No entanto, existem dificuldades para a implantação e uso do TM como tecnologia de acompanhamento do cuidado. Há a necessidade de se trabalhar a aceitação por parte dos profissionais e usuários, esbarrando em fatores socioeconômicos e psicológicos da população, como a dificuldade de interação com a tecnologia, preocupações com a privacidade e preferências por consultas presenciais^(36)^, tal como apontado pelos participantes do presente estudo.

Estudo realizado com idosos apontou desafios a serem enfrentados (por parte dos usuários do serviço e pelos profissionais) para integração de intervenções de TIC no processo de trabalho, como, por exemplo, lacunas de conhecimento, falta de vontade de adotar novas competências, relutância em utilizar tecnologias, conectividade lenta à Internet e a falta de uma política de reembolso adequada. Contudo, a perspectiva positiva dos usuários em relação ao uso das TICs superou os desafios, pois contribuiu com o aumento da motivação, apoio às ações de autogestão, sensação de segurança e redução da ansiedade^(27)^.

Os desafios enfrentados na APS são históricos e envolvem a condição inadequada da rede física das UBS, financiamento insuficiente para a estrutura e investimento em tecnologias, e baixo número e diversificação de recursos humanos. Tais desafios têm dificultado a incorporação de um modelo de cuidado humanizado, como o preconizado pelo MACC^(40)^. Esses desafios também foram citados com frequência pelos profissionais no estudo e limitaram a incorporação do telemonitoramento para a promoção do autocuidado e melhoria na interação com os usuários com condições crônicas.

### Limitações do estudo

Como limitação do estudo, destaca-se o fato de que os participantes são enfermeiros que previamente haviam participado de oficinas de sensibilização para o uso do TM. Essa circunstância pode ter exercido uma influência significativa sobre suas percepções e opiniões sobre o tema em questão, pois o fato de terem recebido informações prévias e orientações específicas sobre o TM pode ter levado a uma predisposição positiva em relação à estratégia.

### Contribuições para a área da Enfermagem, Saúde ou Política Pública

O estudo fornece informações valiosas sobre a percepção dos enfermeiros em relação ao TM na APS e destaca a importância de recursos humanos e estruturais para uma implementação eficaz. Essas informações podem ser úteis no planejamento e orientação de ações voltadas para a melhoria da assistência às pessoas com condições crônicas na APS.

## CONSIDERAÇÕES FINAIS

Os enfermeiros demonstraram interesse na utilização da estratégia de TM, principalmente aqueles que reconheceram sua importância para um acompanhamento frequente e de qualidade. Consideraram que o TM permite manter o contato/ vínculo com o usuário com condições crônicas e/ou familiares, facilita o acompanhamento regular e o fornecimento de orientações importantes, além de promover uma comunicação eficaz, conforme preconizado pelo MACC. Ademais, reduz a necessidade de deslocamentos desnecessários, sem desconsiderar a consulta presencial. No entanto, observaram ser necessário disponibilizar recursos humanos e tecnológicos, assim como reorganizar os processos de trabalho para a viabilidade da implementação do TM na APS de forma eficaz.

Conclui-se, a partir dos achados, que são necessários investimentos em estrutura, tecnologias de informação e recursos humanos, além da continuidade de ações que sensibilizem os profissionais para estabelecer uma assistência utilizando o TM. Ademais, a estratégia deve envolver equipes multidisciplinares, despertando, em cada trabalhador de saúde, o interesse e a participação no tratamento, acompanhamento e avaliação do usuário, além de garantir o fortalecimento da atenção primária, auxiliando no processo de cuidar desses indivíduos.
